# Aerobic Training Modulates the Effects of Exercise-Induced Oxidative Stress on PON1 Activity: A Preliminary Study

**DOI:** 10.1155/2014/230271

**Published:** 2014-10-14

**Authors:** Aneta Otocka-Kmiecik, Marek Lewandowski, Urszula Szkudlarek, Dariusz Nowak, Monika Orlowska-Majdak

**Affiliations:** ^1^Department of Experimental Physiology, Medical University of Lodz, Interdepartmental Chair of Experimental and Clinical Physiology, Mazowiecka Street 6/8, 92-215 Lodz, Poland; ^2^Complex of Vocational Schools, Ozorkow, Poland; ^3^Department of Clinical Physiology, Medical University of Lodz, Lodz, Poland

## Abstract

The aim of the study was to compare the effect of maximal exercise (ME) on paraoxonase (PON) and arylesterase (ARE) activity depending on lifestyle in respect to physical activity. The study was performed on 46 young men divided into two groups: sedentary (S) and physically active (PA). All participants performed ME on a treadmill. PON1 activities, FRAP, uric acid, bilirubin, TBARS, and lipid profile were determined in their blood before, at the bout of, and after ME. No significant differences in PON1 activities were found between S and PA subjects at baseline. Nearly all biochemicals increased at ME in both groups. Both PON and ARE activity increased at the bout of ME in PA subjects and only ARE activity in S subjects. ARE/HDL-C ratio increased at the bout of ME in PA and S subjects. The difference in PON1 activity response to ME between study groups may be a result of adaptation of PA subjects to regular physical activity. We suggest that PON1 activity may be a marker of antioxidant protection at ME and an indicator of adaptation to exercise.

## 1. Introduction

Single strenuous physical activity leads to excessive free radical formation and oxidative stress [1]. However, regular physical activity is known to enhance antioxidant defense mechanisms. Therefore, it is a recognized protective factor against the occurrence and progression of cardiovascular disease. Free radicals produced during each exercise session stimulate adaptive changes in signaling pathways resulting in improved antioxidant mechanisms. Beneficial adaptations at a genetic level are forced [2]. Paraoxonase 1 (PON1) is a part of intrinsic antioxidant system. It is an enzyme (EC.3.1.8.1, aryldialkylphosphatase) associated with HDL. The protective effect of HDL against LDL oxidation is in part attributable to PON1 [3]. There is increasing evidence that reduced activity of PON1 is a predictor of coronary artery disease [4, 5]. The activity of the enzyme is known to depend on many factors, both genetic and environmental [6, 7]. Physical activity was found to modulate PON1 concentration and/or activity [8]. Our previous findings show that physical exercise affects PON1 activity [9, 10]. Yet, some researchers do not confirm these observations [11]. The effect of exercise on PON1 activity may depend on the lifestyle of the examined population and the type and intensity of training. Understanding the role of PON1 in antioxidant mechanisms mobilized at physical activity will perhaps enable the prognosis of remote effects of exercise in sportsmen undertaking physical performance, as well as in subjects with increased risk of atherosclerosis. Hence, the relationship between PON1 activity and physical exercise may have practical implications.

The aim of our study was to compare the effect of maximal exercise (ME) on PON1 activity in young men leading a different lifestyle in relation to their engagement in physical activity.

## 2. Materials and Methods 

### 2.1. Subjects

Research was performed on 46 young men divided into two groups depending on their engagement in physical activity. 20 sedentary (S group) and 26 physically active (PA group) volunteers with an average of 3.5 years of regular training participated in the study. The athletes, students of Vocational Schools of Ozorkow, performed systematic aerobic training with a frequency of 2 hours of exercise 4 times a week. Only nonsmoking participants free of any medication and dietary vitamins with no history of cardiovascular disease, diabetes, and lipid and endocrine disorders were qualified for the study. All volunteers signed a written informed consent prior to enrollment in the experiment. On behalf of minors the consent was signed by their parents. A consent to perform this study was obtained from the Ethics Committee of the Medical University of Lodz on September 26, 2006, License number RNN/163/06/KE.

### 2.2. Study Design

The research was conducted in the air-conditioned Physical Exercise Laboratory at the Interdepartmental Chair of Experimental and Clinical Physiology of Medical University of Lodz from 8.30 a.m. to 11.30 a.m. Men were asked to come after 12 hours of rest and fasting. Before proceeding with ME, basic measurements listed in Table 1 were performed. Bodystat 1500 (Bodystat Ltd., UK) was used to obtain the body composition (lean and fat proportion) and basic metabolic rate (BMR). Then, the first blood sample, named “before,” for biochemical determinations, was drawn. Next, subjects performed ME on a treadmill according to a modified Bruce Protocol [12] until they reached maximal oxygen consumption (VO_2max⁡_) and refused to continue the test due to exhaustion. The ME was monitored using VO2000 MedGraphics Cardiorespiratory Diagnostic Systems, compatible with Breeze Suite 6.2A MedGraphics software. The measured parameters were listed also in Table 1. Reaching VO_2max⁡_ was indicated by achieving respiratory exchange ratio (RER) > 1.1 and the plateau of VO_2_ curve. As the participants reached their VO_2max⁡_, the second blood sample, the “bout” sample, was obtained. Two hours after ME the third blood sample, called “after,” was drawn. The variables listed in Table 2 were determined in the blood samples.

### 2.3. Blood for Assay and Determinations in Plasma

The blood samples were drawn from the median cubital vein into lithium heparin-containing Vacutainer tubes (Becton Dickinson, NJ, USA). The plasma was centrifuged (3000 g, 4°C, 15 min) and stored at −80°C for further analysis. As described by Benzie and Strain, the total plasma antioxidant activity was assayed as ferric reducing activity of plasma (FRAP) [13]. Plasma lipid peroxidation products were determined on the basis of thiobarbituric acid reaction substances (TBARS) as described previously [14]. The concentrations of uric acid (UA), total bilirubin (TBil), and plasma lipids were determined in the Laboratory of University Teaching Hospital number 2 in Lodz, Poland, with Olympus AU 640 autoanalyzer. PON1 plasma activity was measured with two substrates: paraoxon-paraoxonase activity (PON) and phenyl acetate-arylesterase activity (ARE), as described by Nakanishi et al. [15]. Even though the most physiological substrate for PON1 is lactone, the activity of the enzyme towards phenyl acetate was found to highly correlate with lactonase activity and therefore it is widely used [16].

### 2.4. Chemicals

Trizma base was derived from Fluka (Buchs, Switzerland) and Triton X-100 from Serva Feinbiochemica (Heidelberg, Germany), and all other reagents were derived from Sigma Aldrich Chemical (St. Louis, MO, USA).

### 2.5. Statistical Analysis

Results were expressed as mean ± SD. Differences between groups were assessed by Mann-Whitney* U* test. The changes among variables obtained before, at the bout of, and after ME were evaluated using Friedman ANOVA followed by post hoc Wilcoxon matched pairs tests. Pairwise correlations were determined by Pearson's two-tailed bivariate analysis. Significance was set as *P* < 0.05 for all analyses. All statistical tests were performed using Statistica Software v8.

## 3. Results

The comparison of physiological parameters at rest and at the bout of ME in PA and S group revealed pronounced differences in aerobic capacity parameters and body composition. In the PA group, maximal oxygen consumption (VO_2max⁡_), carbon dioxide production (VCO_2_), and maximal oxygen pulse (VO_2max⁡_/HR) were significantly higher while values of heart rate (HR) and body fat were significantly lower (Table 1). The PA group was younger than the S group. However the small age difference did not affect maximal heart rate (HR_max⁡_) of the participants.

### 3.1. Effect of ME on PON1 Activity and Other Biochemical Parameters of Blood in PA and S Subjects

The comparison of the enzyme activity changes in PA and S subjects at the bout of ME revealed a significant increase in both PON1 activities in the PA group and an isolated ARE activity increment in S group (Table 2). Two hours after ME both activities returned to the initial level. There were no significant differences between respective activities in both groups.

The comparison of plasma biochemical parameters in both groups at rest and at ME showed no significant differences between the PA and S volunteers. In both groups a significant increase in all investigated parameters was observed at the bout of ME, with the exception of TBARS value in the S group and serum PON activity adjusted for serum HDL-C level (PON/HDL-C ratio) in both groups. In addition, it was found that the values of FRAP and UA were still increasing 2 hours after ME in both groups.

### 3.2. Correlations of PON1 Activity and Physiological and Biochemical Parameters

No correlations were found between PON and ARE activity and body composition parameters listed in Table 1 in the S and PA groups. Correlating PON and ARE activities with the parameters listed in Table 2 resulted in finding a positive correlation in the PA group between TBARS at the bout and PON before, at the bout of, and after ME (*r* = 0.44, *r* = 0.40, and *r* = 0.42, resp.). Correlations of PON and ARE activities with HDL-C showed a negative correlation in the PA group between ARE at the bout and HDL-C at the bout of and after ME (*r* = −0.44 and *r* = −0.47, resp.). Positive correlations were found between FRAP and UA before, at the bout of, and after ME in the PA group (*r* = 0.59, *r* = 0.70, and *r* = 0.75, resp.) and in the S group (*r* = 0.49, *r* = 0.59, and 0.61, resp.). Furthermore, a positive correlation was detected between FRAP and TBil before ME (*r* = 0.47) in the PA group.

## 4. Discussion

### 4.1. Effect of Aerobic Training on Physiological and Biochemical Profiles of Young Men

The observations of PON and ARE activities were carried out in participants with different physiological profiles in respect to aerobic training, that is, PA and S groups. The manifestation of the distinct lifestyles resulted in a difference in physical exercise capacity in both test groups such as HR at rest and VO_2max⁡_, VO_2max⁡_/HR, and VCO_2_ at ME (Table 1). Biochemical parameters did not differ between groups either at rest or at ME. These values are very similar to those obtained in our previous studies [9, 10]. However, there has been some tendency (but no statistically significant difference) to a higher antioxidant defense (higher FRAP, UA, and TBil) and lower lipid oxidation (lower TBARS), as well as a more favorable lipid profile (lower TChol, LDL-C, and TG and higher HDL-C) in the PA group (Table 2). Our observations also show that there is a tendency for higher values of PON and ARE activities in PA in comparison to S participants. Therefore, PON1 may provide a higher antioxidant defense in physically active subjects. Yet the discrepancy between groups, though considerable, is not significant, perhaps due to a large, at least a 40-fold [17], variation in serum PON1 activity among individuals and a relatively small group of subjects. In some studies PON1 activity was found to be higher in regularly training subjects in comparison to sedentary controls [18]; yet others reported no influence of regular exercise on basal PON1 activity levels [19]. These discrepancies in observations may result from the different type, intensity, frequency, and period of training. We have carried out tests on young athletes on the verge of their sports career, in which the beneficial effects of regular physical activity may be just beginning to be revealed.

### 4.2. Effect of ME on Oxidoreductive Balance of the Blood in PA and S Subjects

Single ME resulted in a significant increase in all designated biochemical parameters in the plasma of both groups with one exception: in the S group TBARS increase was not significant (Table 2). However, this effect may be due to high SD in the S group, higher than that in the PA group. It may account for a high interindividual variation of exercise tolerance in subjects with low adaptation to strenuous physical activity. In a table by Vollaard et al. (2005), in the plasma of trained and untrained subjects at rest and during exercise of varying intensity, the TBARS levels rise, fall, or remain unchanged in response to physical exercise [2]. In our study both FRAP and UA continued to rise 2 hours after ME. Similar results on UA changes were described in other studies [9, 10, 20]. Additionally, Yanai and Morimoto (2004) found that the serum urate levels were significantly elevated even 3 weeks after exhaustive training [21]. The observed convergence of UA and FRAP is as expected, as UA is known to secure 2/3 of the antioxidant defense in human plasma [22]. In our study, UA was found to participate in total antioxidant capacity regardless of the training status. TBil correlated with FRAP only in PA subjects, which is a mechanism of adaptation to regular physical training.

### 4.3. Effect of ME on PON1 Activities of the Blood in PA and S Subjects

Observation of PON1 activity changes at the bout of ME revealed significant increases in both activities (PON and ARE) in the PA group and an increment of only ARE, but not PON, in the S group (Table 2). Tomás et al. (2002), who determined selectively PON activity, also showed that it increased substantially at the bout of exercise [23]. However, PON activity increment was observed in both: in a group which performed regular training for 16 weeks prior to testing and in a group which remained sedentary in the pretesting period. Yet, Tomás et al. found that the elevation in PON activity after regular training was substantially higher than after a sedentary lifestyle [23]. A more complex response to ME in PA subjects (increase in both PON1 activities) than in the S subjects (increase in only ARE activity) in our study suggests that regularly trained men have a more efficient adaptation to oxidative stress. In the light of the observed large standard deviation of PON1 activity, obtaining a significant increase in PON1 activities at the bout of ME reinforces our conclusion that physical exercise causes PON1 enzymatic activation. Moreover, in our study we observed positive correlation of TBARS and PON only in the PA group but not in the S group. It may also be a manifestation of adaptation to regularly repeated physical activity, as increased lipid peroxidation mobilizes higher antioxidant protection and PON activation.

We therefore conclude that this enzyme is a substantial part of antioxidant defense during exercise and we suggest that it is a component of adaptive processes in regularly trained men.

### 4.4. Correlations of PON1 Activity and Physiological and Biochemical Parameters

The lack of correlation between PON and HDL-C found in our study was also described by some groups [23], though others reported positive correlations between these parameters [24]. The negative correlation of ARE activity and HDL-C in the PA group, as revealed in this study, was quite unexpected. It would seem reasonable that PON1 activity should increase with the increment of HDL concentration, since the enzyme particle is bound to HDL [25]. Interestingly, there was no correlation of ARE activity and HDL-C before ME. The negative correlations were found only at the bout of ME and after ME, when HDL may have been modified due to oxidative stress at ME. Native HDL particles were reported to stimulate PON1 activity. Apolipoproteins associated with HDL, especially apolipoprotein A-I, are essential for optimal PON1 function [26]. However, not all HDL particles have a similar reinforcing effect on PON1 activity. As a matter of fact, oxidative modification of HDL was shown to reduce PON1 activity measured as ARE [27]. Also, HDL subpopulations were found to exhibit different properties, which can alter the activity of PON1, especially the properties of the active center of the enzyme [28]. Dullaart et al. reported that HDL-C correlated positively with large and medium HDL particles and inversely with small HDL particles [24]. Additionally, we performed analyses of PON/HDL-C and ARE/HDL-C ratios at ME. We found that ARE/HDL-C ratio increases at the bout of ME in both groups, which suggests that ARE activity increment is more pronounced and disproportionate to HDL-C increase. These results confirm that the increment in ARE activity at the bout of ME is not a direct result of HDL-C rise.

## 5. Conclusions

We conclude that ME mobilizes PON1 as part of antioxidant defense. The increment of PON1 activity at ME is more pronounced and complex in PA than in S individuals. It may be an effect of adaptation to regular training and an increase of intrinsic antioxidant system response to oxidative stress. This is our third study in which we have observed an increase in PON1 activity at the bout of ME. Therefore, we propose that the enzyme may be considered one of the antioxidant protection markers at ME.

## Figures and Tables

**Table 1:** Physiological measurements of the study groups (mean ± SD).

Measurements	Sedentary group (S)	Active group (PA)
	(*n* = 20)	(*n* = 26)
Before exercise		
Age (years)	21 ± 1	17 ± 1^*^
Height (cm)	180 ± 8	177 ± 7
Weight (kg)	77 ± 14	69 ± 8
Body fat (%)	17 ± 4	12 ± 6^*^
Lean (kg)	64 ± 13	61 ± 9
BMI (kg/m^2^)	23 ± 3	22 ± 2
BMR (kcal)	1955 ± 298	1877 ± 196
HR (beats/min)	74 ± 13	66 ± 12^*^

At the bout of maximal exercise		
VO_2max _(mL/min)	3364 ± 934	4304 ± 791^*^
VO_2max_ (mL/kg/min)	46 ± 7	61 ± 10^*^
VCO_2_ (mL/min)	3872 ± 1126	5038 ± 782^*^
VO_2max_/HR (mL/beat)	20 ± 5	26 ± 10^*^
HR_max_ (beats/min)	191 ± 9	181 ± 35
RR (br/min)	52 ± 7	54 ± 10
VE (L/min)	122 ± 30	127 ± 21
RER	1.16 ± 0.1	1.18 ± 0.08

BMI: body mass index, BMR: basic metabolic rate, HR: heart rate, VO_2max_: maximal oxygen consumption,VCO_2_: maximal carbon dioxide production, VO_2max_/HR: maximal oxygen pulse, HR_max_: maximal heart rate, RR: respiratory rate, VE: minute ventilation, and RER: respiratory exchange ratio.

∗Statistical significance of difference between S and PA groups, *P* < 0.05.

**Table 2:** Biochemical measurements at maximal exercise (mean ± SD).

Biochemical measurements	Before		Bout		After	
	Sedentary group (S) (*n* = 20)	Active group (PA) (*n* = 26)	Sedentary group (S) (*n* = 20)	Active group (PA) (*n* = 26)	Sedentary group (S) (*n* = 20)	Active group (PA) (*n* = 26)
PON (U/L)	340 ± 268	435 ± 320	363 ± 269	489 ± 319^*^	370 ± 269	435 ± 315
ARE (U/mL)	133 ± 61	141 ± 52	183 ± 91^*^	191 ± 93^*^	147 ± 54	152 ± 74
FRAP	1.1 ± 0.2	1.3 ± 0.2	1.2 ± 0.2^*^	1.5 ± 0.3^*^	1.4 ± 0.2^*^	1.6 ± 0.4^*^
TBARS (mM/L)	3.4 ± 1.4	3.1 ± 1.5	3.7 ± 2.1	3.6 ± 1.8^*^	3.1 ± 1.2	3.0 ± 1.3
UA (mM/L)	0.32 ± 0.06	0.33 ± 0.07	0.34 ± 0.06^*^	0.36 ± 0.07^*^	0.39 ± 0.07^*^	0.40 ± 0.07^*^
TBil (*μ*M/L)	15.6 ± 8.5	17.4 ± 7.2	16.8 ± 9.2^*^	20.4 ± 8.4^*^	15.5 ± 9.6	18.7 ± 8.2
TChol (mM/L)	4.6 ± 0.9	4.2 ± 0.9	5.0 ± 1.1^*^	4.5 ± 0.9^*^	4.6 ± 1.0	4.2 ± 0.9
HDL-C (mM/L)	1.2 ± 0.2	1.3 ± 0.2	1.3 ± 0.2^*^	1.4 ± 0.2^*^	1.2 ± 0.2	1.3 ± 0.2
LDL-C (mM/L)	2.8 ± 0.6	2.5 ± 0.7	3.0 ± 0.8^*^	2.6 ± 0.8^*^	2.8 ± 0.6	2.4 ± 0.9
TG (mM/L)	1.4 ± 1.0	0.9 ± 0.3	1.5 ± 1.0^*^	1.1 ± 0.5^*^	1.3 ± 1.1	1.1 ± 0.5
PON/HDL-C	302 ± 239	335 ± 294	300 ± 225	359 ± 277	327 ± 236	353 ± 337
ARE/HDL-C	114 ± 46	107 ± 45	148 ± 78^*^	144 ± 103^*^	126 ± 37	114 ± 60

Biochemical measurements were performed in the plasma obtained from sedentary (S) and active (PA) subjects before, at the bout of, and after maximal exercise on treadmill.

FRAP: ferric reducing activity of plasma, TBARS: thiobarbituric acid reaction substances, UA: uric acid, TBil: total bilirubin, TChol: total cholesterol, HDL-C: high density lipoprotein cholesterol, LDL-C: low density lipoprotein cholesterol, TG: triglycerides, PON: paraoxonase activity, and ARE: arylesterase activity; PON/HDL-C ratio; ARE/HDL-C ratio.

∗Statistical significance of difference versus respective value “before,” *P* < 0.05.

All differences between S and PA subjects were not statistically significant.
